# Regional electrical structure of the Andean subduction zone in central Chile (35°–36°S) using magnetotellurics

**DOI:** 10.1186/s40623-017-0726-z

**Published:** 2017-10-12

**Authors:** Valentina Reyes-Wagner, Daniel Díaz, Darcy Cordell, Martyn Unsworth

**Affiliations:** 1 Departamento de Geofísica, Universidad de Chile, Blanco Encalada 2002, Santiago, Chile; 2 Centro de Excelencia en Geotermia de Los Andes, Plaza Ercilla 803, Santiago, Chile; 3 Department of Physics, University of Alberta, Edmonton, AB T6E 2E1 Canada

**Keywords:** Magnetotellurics, Volcanism, Southern Volcanic Zone, Deformation, Subduction zone

## Abstract

A profile of broadband magnetotelluric stations was acquired between 2009 and 2016 at 35°–36°S in the Southern Volcanic Zone of the Chilean Andes to image the subduction zone and its relation with the volcanic arc at this latitude. This transect extends from the Coastal Cordillera across the Central Valley and the volcanic arc of the Principal Cordillera to the Argentine border. Two active volcanic complexes are found along this profile: Tatara-San Pedro is located on the modern volcanic front, and the Laguna del Maule volcanic field is found approximately 30 km to the east. The latter exhibits considerable signs of unrest, such as uplift rates of up to 25 cm/year, and has produced a high concentration of silicic eruptions in the last 25 ky. The data covered the period range from 0.001 to 1000 s. Robust processing techniques were used, including remote reference, and dimensionality was investigated by estimation of geoelectric strike, skew and analysis of the induction arrows. The data were modeled using a 2D inversion algorithm to produce a resistivity model which was consistent with surface geology and seismicity. The final resistivity model shows a generally resistive fore-arc structure, coincident with the tectonic environment, and a wide conductive region from the volcanic front to the east. This suggests a broad region of magmatism throughout the arc, related to three distinct magma bodies, associated with the Tatara-San Pedro and Laguna del Maule volcanic complexes and the Mariposa Geothermal System.

**Electronic supplementary material:**

The online version of this article (doi:10.1186/s40623-017-0726-z) contains supplementary material, which is available to authorized users.

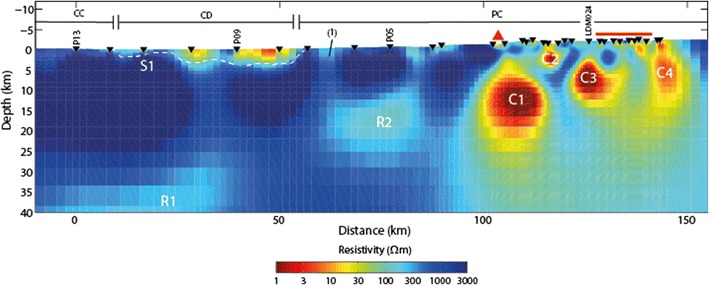

## Introduction

The Southern Volcanic Zone (SVZ) of the Andes extends between 33° and 46°S and has been formed as the Nazca Plate subducts obliquely beneath the South American Plate in a direction of approximately N78°E (Somoza 1998). The SVZ is characterized by many active volcanoes located in the well-developed volcanic arc. As in other subduction zones, fluids released from the slab facilitate partial melting in the upper mantle by reducing the melting point of the rocks (Grove et al. 2012). These mafic melts then rise due to buoyancy and differentiate on their way to the surface where they are erupted (Hildreth and Moorbath 1988).

Understanding the behavior of melts in a subduction zone requires geophysical imaging. The electrical resistivity of crustal and upper mantle rocks is sensitive to the presence of both aqueous fluids and partial melts. The resistivity can be measured from the surface using magnetotellurics (MT)—a passive geophysical technique that uses natural electromagnetic signals to image subsurface resistivity. A number of previous MT studies have imaged subduction zones worldwide, including the Central and Southern Andes (Brasse and Soyer 2001; Brasse and Eydam 2008; Brasse et al. 2009; Diaz et al. 2012; Comeau et al. 2016). One common result from these studies is that the resistivity structure shows significant along strike variations. In the Central Volcanic Zone, which extends from 14° to 27°S, deep conductive features are found below the Altiplano and Puna plateaus, approximately 100 km east of the modern volcanic arc (Brasse and Eydam 2008; Diaz et al. 2012; Comeau et al. 2016). Studies at 38°–40°S in the central SVZ have established that deep conductors are associated with fault zones, particularly the Liquiñe-Ofqui fault zone at the volcanic arc which is a trench-parallel structure between 38° and 47°S that may control the location of eruptive centers in this area (Brasse and Soyer 2001; Brasse et al. 2009). Although the region of the 2010 M8.8 Maule earthquake, located in the SVZ, has been relatively well studied with seismic measurements (e.g., Pesicek et al. 2012), it has not yet been imaged using magnetotellurics.

One of the most important volcanic features in this area is the Laguna del Maule (LdM) volcanic field which is currently undergoing one of the highest rates of deformation measured at a volcano that is not actively erupting. It has reached uplift rates of 25 cm/year and accumulated a maximum vertical displacement of at least 1.8 m since uplift was first detected in 2007 (Feigl et al. 2014; Le Mével et al. 2015). The LdM volcanic field is also the location of the greatest concentration of post-glacial rhyolite in the Andes (Singer et al. 2014). Geophysical data support the existence of a magma body below LdM, with the source of surface deformation attributed to an inflating magma sill, although the depth is still a subject of debate between different geophysical studies (Le Mével et al. 2016; Miller et al. 2017; Cordell et al. 2016). The addition of new magma into the reservoir has been related to seismic swarms and gravity changes observed in the area (Singer et al. 2014; Miller et al. 2017).

While detailed geophysical studies have been made of the magma body beneath Laguna del Maule, the regional context is poorly understood. The goal of this paper is to present a regional transect which relates the regional structure of the subduction zone to the shallow magma bodies beneath this area of the Andes.

## Geological context

**Fig. 1:**
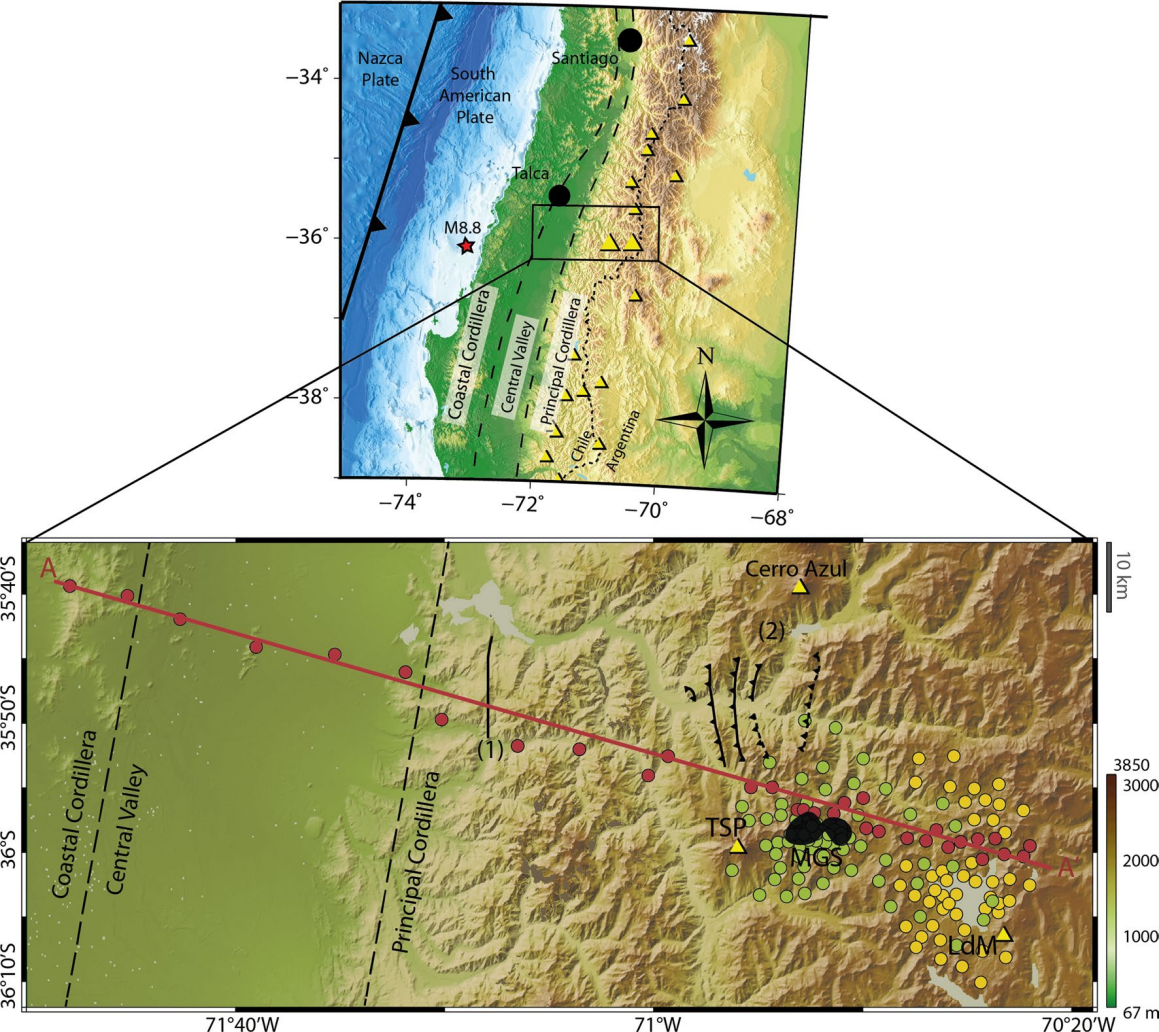
Study area. A regional map shows the morphostructural units delimited by black dashed lines and volcanoes are represented by yellow triangles, the volcanic arc is defined by the location of these volcanoes. The location of the 2010 M8.8 Maule earthquake epicenter is shown as a red star. A map inset shows the MT study area. Broadband MT stations are shown in circles: green represents those measured by Alterra Power (2009–2012), yellow by Universidad de Chile and University of Alberta (2015–2016). The red circles are the stations used in the 2D inversion. The profile used in the 2D inversion is denoted by AA′. MGS is delimited in black by the MT anomaly from Hickson et al. (2011). The faults shown are (1) fault mapped by Sernageomin (2003) using surface features and (2) reverse east-verging faults, from west to east: García Fault, Las Corrientes Fault and Hornitos Fault (Astaburuaga 2014)

At this latitude, the Andean subduction zone is composed of three morphostructural units (Fig. 1), from west to east these are: the Coastal Cordillera (CC), Central Valley (CV), and Principal Cordillera (PC). Along the western flank of the Coastal Cordillera, it is possible to find the oldest Paleozoic rock outcrops of the study area, which have acted as a barrier to deposition of volcano-sedimentary sequences present in the Central Valley between the Coastal Cordillera and the Principal Cordillera. In contrast, the eastern flank of the Coastal Cordillera is primarily characterized by Mesozoic intrusive and volcano-sedimentary sequences, reflecting the eastward migration of the volcanic arc since the Jurassic. The Central Valley consists of a depression filled with Quaternary alluvial and volcanic deposits. The Principal Cordillera consists mainly of Cenozoic rocks from the Abanico Formation (volcano-sedimentary sequences) deformed by folds and thrusts, and the Cola de Zorro Formation composed of intrusive rocks from the Cenozoic and volcanic sequences from the active volcanic arc (Sernageomin 2003; Astaburuaga 2014). Some of the major structures found in the Abanico Formation are shown in Fig. 1.

Focusing on the active volcanic arc in the region of this study, there are two important features: the Tatara-San Pedro (TSP) and Laguna del Maule complexes. The TSP complex is located at 36°S on the volcanic front of the SVZ. Volcanism at this complex features lavas ranging from primitive basalt to high silica rhyolites, with basaltic andesitic lavas being the most common product (Singer et al. 1997; Dungan et al. 2001). It comprises eight or more volcanic sequences (Dungan et al. 2001) and at least three central vents regions (Singer et al. 1997) during 930 ky of activity. San Pedro volcano is the youngest of the TSP complex, with basaltic to dacitic eruptive activity during the Holocene. Petrological evidence reflects several intrusions of basalt during the Holocene and the past 350 ky (Costa and Singer 2002).

Approximately 30 km to the east of the modern volcanic front, the Laguna del Maule (LdM) volcanic field is a basaltic to rhyolitic system active since the Pleistocene, which includes the formation of the Bobadilla Caldera at 950 ky. Post-glacial volcanism (< 25 ky) is characterized by the lack of basaltic products and the eruption of 36 rhyodacitic to rhyolitic lavas and domes from 24 vents distributed around the LdM basin, giving a total volume of 6.4 km^3^ (Hildreth et al. 2010). Petrological evidence supports the hypothesis of mafic intrusions into the upper crust over the last 26 ky (Andersen et al. 2017).

Related to both of these complexes is the Mariposa Geothermal System (MGS), located between LdM and the TSP complex. Several geological and geophysical studies have been performed in the area to assess the geothermal potential of the reservoir, indicating a high enthalpy geothermal system. This includes an MT survey carried out inside the Laguna del Maule and Pellado geothermal concessions between 2009 and 2012 (Hickson et al. 2011). Some of this MT data have been used in the current study.

## Data acquisition and processing

To extend the detailed study at LdM into a regional transect, 20 broadband MT stations were acquired along a profile perpendicular to the trench at this latitude (profile direction N100°E), from the Coastal Cordillera, across the Central Valley and the volcanic arc to the Argentine border (Fig. 1). Data of these 20 stations were collected during the 2015–2016 field seasons using Metronix ADU-07 data loggers with MSF-07 induction coil magnetometers and EFP-06 (Pb-PbCl) electrodes. An additional 12 broadband MT stations were used from previous MT field work in 2009–2012. Looking for a good trade-off between resolution, investigation depth, and measurement time, a period band between 0.001 and 1000 s was chosen for data acquisition, with an average station spacing of 10 km (see Fig. 1) and measurement times between 15 and 24 h per site. Robust data processing techniques were applied, using the method of Egbert and Booker (1986) in a first stage. Then 15 of the 20 stations were reprocessed by the company CGG electromagnetics to obtain better responses with the code of Larsen et al. (1996). All of the apparent resistivity and phase curves and their fit to the final inversion model response are shown in Additional file 1: Figs. 1 and 2.

Sites located in the Coastal Cordillera show apparent resistivity and phase curves that are consistent with an ocean effect at longer periods as the TM apparent resistivity increases and TE apparent resistivity decreases, while TM and TE phases present values of less and more than 45°, respectively (P13 in Fig. 5). Those located in the Central Valley are affected by local electromagnetic noise from power lines, in most cases data around the dead band had to be removed; however, curves are consistent with a low-resistivity surface layer (P09 in Fig. 5). In this case, remote reference processing was used when possible, improving apparent resistivity and phase curves. In the volcanic arc, apparent resistivity values decreases with period showing consistent behavior with a deep high-conductivity structure (LDM024 in Fig. 5).

**Fig. 2:**
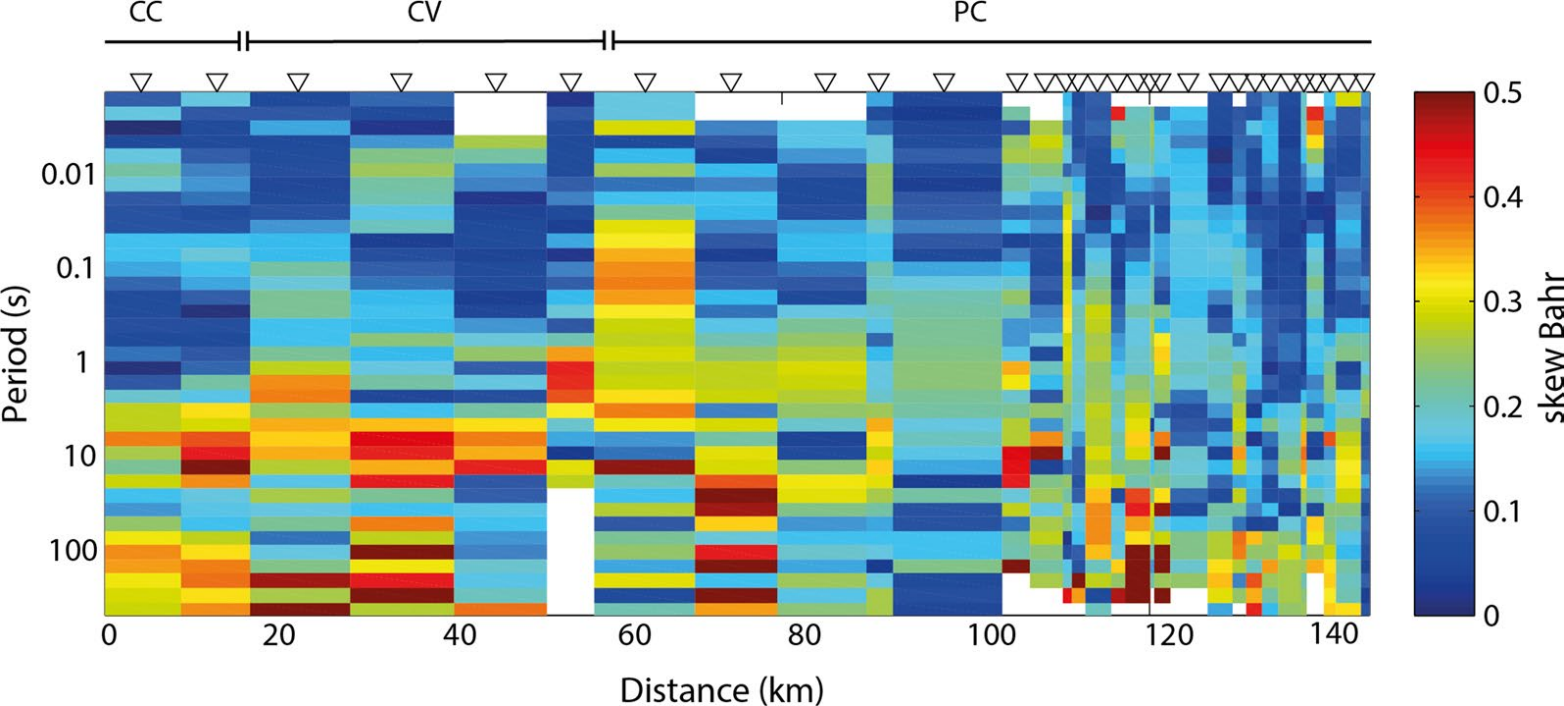
Profile showing skew values according to Bahr (1988). Each column represents an interpolation of the skew value between two sites. The width of the columns considers the spacing between the sites along the profile, resulting in a more detailed image of this parameter to the East. Low skew (< 0.3) indicates relatively 2D structure

Before inverting the data, the dimensionality of the impedance data was investigated. The skew of the impedance tensor was calculated according to Bahr (1988), and this is shown in Fig. 2. The closer the value of the skew is to zero, the closer the data approximate an ideal 2D geoelectric structure, while values above 0.3 may be an indicator of 3D regional structures. As shown in Fig. 2, skew values are mostly lower than 0.3, supporting the assumption of a generally 2D structure. Larger values are seen, especially at longer periods at the sites located on the CC, CV and western PC (i.e., the western side of the profile). Because of the cultural noise present on these measurements, the real value of the skew at those sites and frequencies is uncertain, as it could be altered by the low quality of the data. Large skew values are also seen at some sites located on the eastern PC for periods longer than 1 s, which could be related with the presence of 3D structures in that area.

**Fig. 3:**
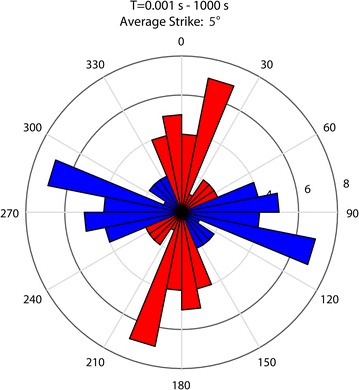
Rose diagram showing the geoelectric strike direction for 0.001–1000 s for stations used in 2D inversion using the strike decomposition program of McNeice and Jones (2001)

The geoelectric strike angle was calculated (Fig. 3) using the tensor decomposition algorithm of McNeice and Jones (2001), which solves simultaneously for the best-fitting strike at multiple sites and multiple frequencies. The average strike obtained with the full data set (all stations, all periods) was approximately N5°E, but when considering different frequency bands separately, the results vary. At higher frequencies the strike was not well constrained, while for longer periods (> 1 s) the results were more coherent and gave a strike value close to N–S direction (~ N0°E). With a clear regional geoelectric strike for longer periods, a 2D resistivity structure perpendicular to that direction can be assumed on a regional scale; therefore, 2D modeling is a valid approach for this kind of study. As the rotation obtained by this analysis was very small (< 5°), the inversions were carried out with the original unrotated data.

The dimensionality was also investigated using induction arrows, primarily to test the existence of conductive structures off-profile. These were calculated according to the convention of Wiese (1962) and are shown for characteristic periods in Additional file 2: Fig. 1. For short periods, the arrows do not show any consistent pattern, probably due to local, shallow 3D features. At mid-range period, the induction arrows show some off-profile conductors at specific sections of the profile, which are described in Additional file 2: Fig. 1. For longer periods (> 64 s) the induction vector data are not consistent with the geoelectric strike as they have a large N–S component. In the ideal case, a N–S geoelectric strike should result in E–W-oriented induction arrows. Similar phenomena were observed further south in the SVZ (38°–39°S) by Brasse et al. (2009). This was interpreted to be the result of an anisotropic layer in the lower crust, which could also be affecting the deepest part of the data in this region (35°–36°S).

The data were then inverted using the method of Rodi and Mackie (2001), a nonlinear conjugate gradients (NLCG) algorithm for 2D inversion of MT data. The preferred resistivity model (Fig. 5) was obtained by jointly inverting the TM and TE modes. Even though the conductive bodies found using the tipper data are located in the same positions and their upper limits are at the same depths, their depth extent is larger than one could expect. This is likely due to treating 3D structures with a 2D approach. The initial resistivity model was a 100 Ωm halfspace using the stations altitude data for topography, even though the topography, especially at the volcanic arc, is 3D. This approximation could have an impact on the anomaly pattern beneath the Principal Cordillera. The ocean and oceanic subducting plate were represented as 0.3 and 1000 Ωm bodies, respectively (Worzewski et al. 2011; Brasse et al. 2009). The resistivity of the ocean was fixed and the bathymetry included, while the top of the oceanic plate was based on the Slab 1.0 model (Hayes et al. 2012), and its resistivity was allowed to change during the inversion.

**Fig. 4:**
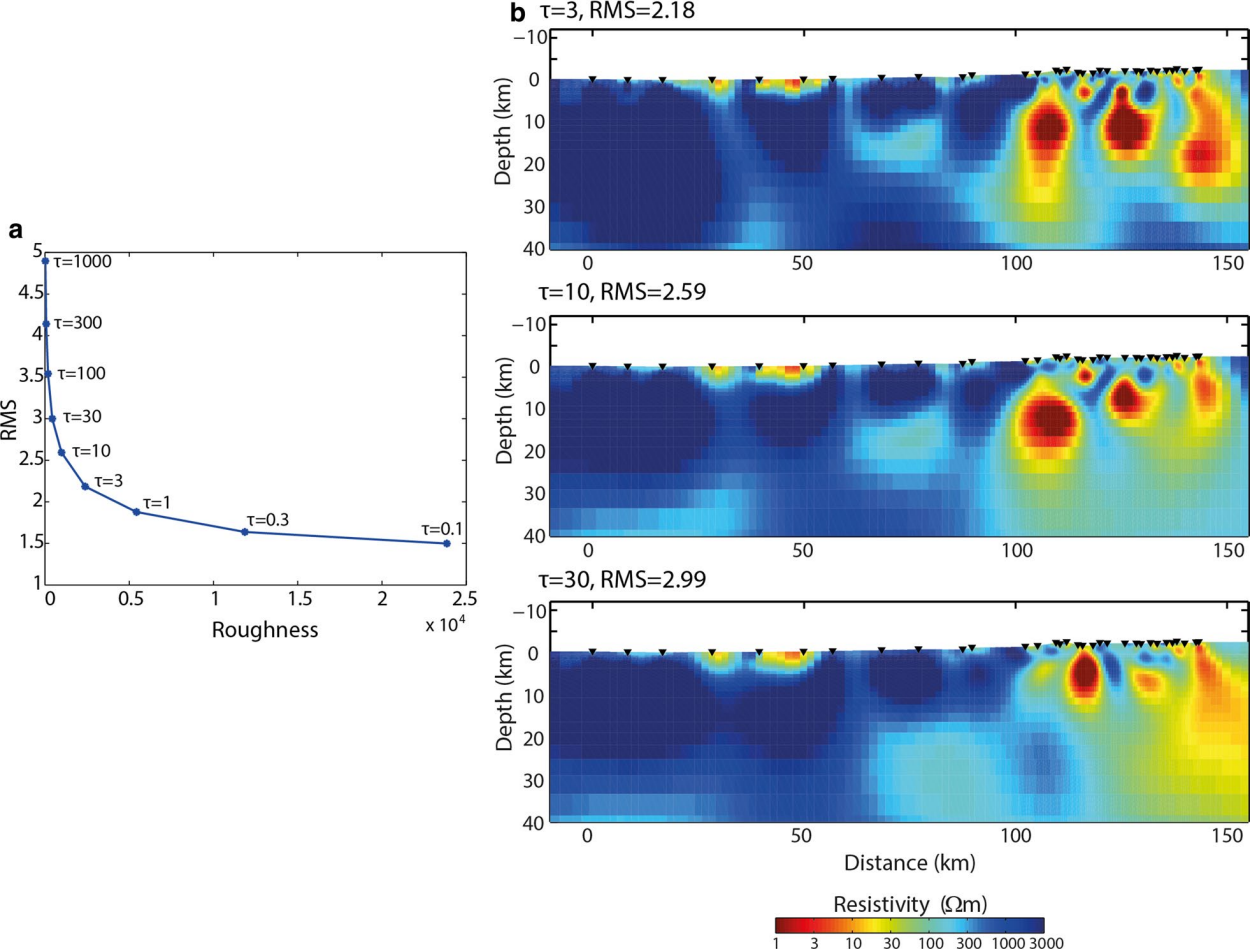
Determination of *τ* value. **a** L-curve for different *τ* values ranging from 0.1 to 1000. **b** 2D inversion models obtained with *τ* = 3, *τ* = 10 and *τ* = 30

An L-curve (trade-off) analysis was performed using smoothing parameters from *τ* = 0.1 to *τ* = 1000. Based on this and the resulting models, *τ* = 10 was selected for this inversion, considering the smoothness of the model obtained, a small change in the RMS, and that the general features of the model are consistent between the inversions with most other *τ* values (see Fig. 4). Error floors used in the final model were 10% for apparent resistivity and 5% for phase, and static shifts were included as an inversion parameter for both modes. The final root-mean-square (RMS) obtained for the inversion was 2.59 achieved after 200 iterations.

## Results and discussion

**Fig. 5:**
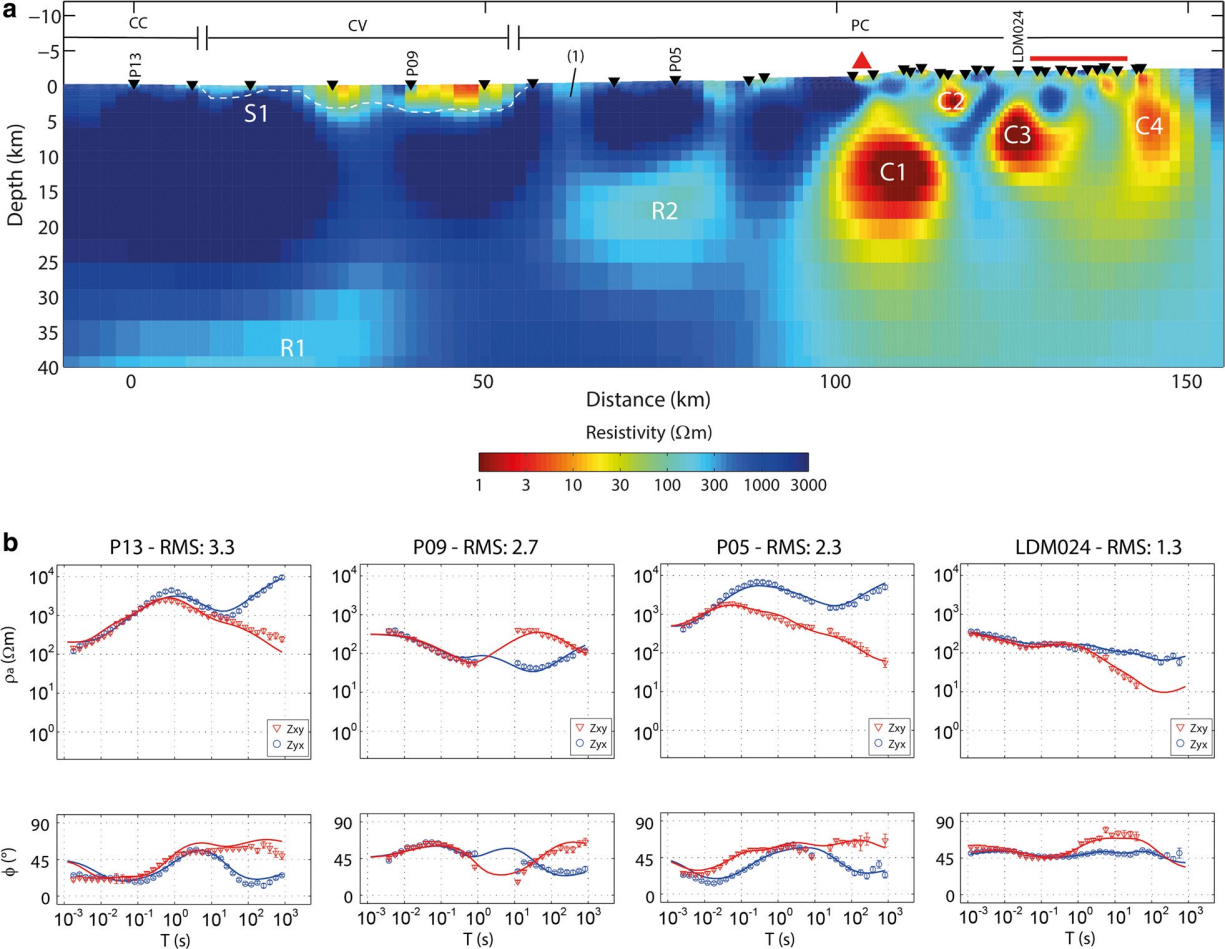
2D inversion results. **a** Preferred resistivity model using *τ* = 10 and total RMS = 2.59. Resistivity features S1, R1, R2, C1, C2, C3, and C4 are explained in the text. Black triangles correspond to MT stations. Morphostructural units delimited: Coastal Cordillera (CC), Central Valley (CV) and Principal Cordillera (PC). (1) Corresponds to the same fault shown in Fig. 1. The TSP complex is represented by a red triangle and the LdM complex by a red rectangle. **b** Apparent resistivity, phase and model response for several stations

The inversion model shows four principal conductive features (C1, C2, C3, and C4; Fig. 5) all located on the eastern side of the profile (i.e., from the volcanic front to the east). The western side, or fore-arc region, shows high resistivity values in general, with the exception of a low resistivity structure located in the Central Valley, between 10 and 50 km along profile and a discontinuity at around 60 km along profile, both at shallow depths (< 5 km). The Pacific Ocean and Nazca plate are not shown in Fig. 5, although they were included in the final resistivity model. Including these structures at the western edge of the model leads to less extreme resistivity values especially under the CV to the west.

### Volcanic arc

As mentioned before, high electrical conductivity can be expected in active volcanic areas and may be due to partial melt, aqueous fluids, and hydrothermal alteration—or some combination of these factors. High-conductivity values were also observed below the volcanic arc at the long period MT profiles from Brasse and Soyer (2001) and Brasse et al. (2009) south of the present profile, but these anomalies were located at greater depths than in this study. Because of the different methodology used in these studies, the resolution at depth varies, and therefore, the comparison between the two models cannot be made directly. A better comparison will be possible with the long period data that will be collected in the study area in the near future.

However, the conductive anomalies found in the southern profiles were interpreted as related to the Liquiñe-Ofqui fault zone, while in this case the three conductive anomalies that demarcate the volcanic arc can be related to different volcanic or geothermal systems.

C1 is spatially correlated with the active volcanic front, in this case with the TSP volcanic complex located south of the profile. Considering the most recent lavas of the TSP complex, young Holocene dacites, Costa et al. (2004) constrained pre-eruptive conditions as 200 ± 50 MPa, 850 ± 10 °C, and 4.5–5.5 wt% H_2_O in the melt. SiO_2_ content ranges between 51 and 66%, while Na_2_O is between 3.23 and 4.56% (Costa et al. 2004). Using these values, the estimated resistivity of a magma reservoir melt would range between 2 and 10 Ωm (Pommier and Le-Trong 2011)

To the east, the MGS was previously outlined by a low-resistivity horizontal layer with depths of approximately 500 m using 3D modeling (Hickson et al. 2011). This is interpreted as a smectite clay alteration cap formed over the active geothermal system (Hickson et al. 2011). High enthalpy geothermal systems, such as Mariposa, occur where magma intrusions reach shallow depths (< 10 km), acting as a heat source for the hydrothermal system (Berktold 1983; Spichak and Manzella 2009). In the 2D resistivity model (Fig. 5), C2 is a low-resistivity anomaly found below the clay cap at a depth of 2 km, and thus it is interpreted as part of the MGS, likely acting as the heat source.

The resistivity model obtained by Cordell et al. (2016), with a 3D MT inversion around LdM contains four main conductive features, with the deepest ones to the northwest of the inflation center. C3 is consistent in location along profile and approximately in depths with these conductors, even though the model obtained here is 2D. The location of C3 near the 19 ka Los Espejos rhyolite suggests that this anomaly is associated with a magmatic body, probably rhyolitic due to the last eruptions in the area. Resistivity values found at this conductive body (< 5 Ωm) are consistent with a rhyolitic magma at 200 MPa, a temperature between 760° and 875 °C and water content from 1 to 6 wt% (Gaillard 2004), conditions that are similar to the ones found by Andersen et al. (2017) for the Los Espejos eruption at LdM.

Other geophysical methods have imaged a magma body beneath LdM, but are all localized studies extending to a maximum depth of 5 km (Le Mével et al. 2016; Miller et al. 2017), therefore they are not capable of imaging a body at greater depths such as C3. However, these localized studies support the hypothesis that the shallow reservoir is being fed by magma originating from greater depths. C3 could serve as possible source of material for this shallow reservoir.

Although the local 3D resistivity model of Cordell et al. (2016) does show some conductive bodies at the eastern side of LdM, these are smaller than the conductive body C4 found in this study. Considering that this anomaly is located at the edge of the profile, C4 is treated as an artifact caused by the 2D inversion of 3D data in this area (Cordell et al. 2016) and is not interpreted geologically. Other than C4, all other conductive features were validated by sensitivity tests (see Additional file 3: Figs. 1, 2 and 3), considering the data fit of the apparent resistivity and phase curves for observed and modeled data.

The 2D resistivity model contains a number of conductors which are all to the east of the active volcanic front. This model is interesting for a number of reasons since it suggests that magmatism occurs in a broad region of the arc. The absence of conductors west of the volcanic arc may also be significant, in light of the fact that the magmatic arc is migrating eastward. The shallow magma bodies that feed eruptions generally take tens to hundreds of thousands of years as a crystalline mush before being remobilized briefly before erupting (Annen et al. 2015), and hydrothermal systems may also be present after the stop of new magma supply. Thus if the volcanic arc has been migrating uniformly eastward, it would be expected that some low-resistivity zones would be observed to the west of the present location of the volcanic arc. The absence of such features could be explained if the volcanic arc has jumped to the present location, rather than moving uniformly.

### Fore-arc

The fore-arc resistivity structure at shallow depths is consistent with the observed superficial geology (Sernageomin 2003). The surface conductor S1 delimited by the white dashed line is consistent with the location of the Central Valley. Resistivity values are lower than 300 Ωm, associated with young sediments and sedimentary rocks and weathered basement rock. Also, the higher resistivity values (> 1000 Ωm) in the near surface persist at depth and are consistent with older intrusive and volcano-sedimentary sequences associated with the Coastal Cordillera and Principal Cordillera along the west and east sides of the valley, respectively.

The discontinuity at a distance of 60 km on the profile correlates with a fault identified by surface geology studies in the western Principal Cordillera ((1) in Fig. 5), which could enhance the infiltration of meteoric water, presenting lower resistivity values than the volcano-sedimentary sequences in the surroundings. Decreased resistivity values have been detected in other fault zones such as the San Ramon Fault at 33.5°S (Díaz et al. 2014) and the Liquiñe-Ofqui Fault in southern Chile (Held et al. 2016).

At greater depths, resistivity values range from 1000 Ωm to more than 3000 Ωm. Two interesting features are found here, the first below the Coastal Cordillera and Central Valley at 35 km depth (R1 in Fig. 5) and the second between 10 and 25 km below the western Principal Cordillera (R2 in Fig. 5). Although somewhat resistive (between 100 and 300 Ωm), these bodies present a high contrast with the much higher-resistivity surroundings. The requirement of these features in the resistivity model was validated by sensitivity tests (see Additional file 3: Figs. 5 and 6). Model feature R1 is consistent with the serpentinized mantle wedge according to Farías et al. (2010), but a deeper study is needed to investigate its relation with the subducting slab. Farías et al. (2005, 2010) proposed a ramp-flat structure that connects the subduction zone with the mountain belt in northern and central Chile and suggest that this structure could also be present in south central Chile. The anomaly R2 could be related to this ramp-flat structure due to the seismicity produced there.

## Conclusions

In contrast with other MT studies in the Andes mentioned above, the resistivity model derived from this new profile shows a region of high conductivity in the upper crust below the volcanic arc, starting from the modern volcanic front and extending at least 40–50 km to the east.

Even though the resistivity model presented here is 2D, it agrees with local3D inversion model derived around the LdM volcanic field. The feature C3 is consistent in location and depth with the conductive features identified with the 3D inversion mentioned above. This conductive body is interpreted as a possible source of material for the observed inflation at LdM. The other high-conductivity areas (C1 and C2) are coincident with an active volcano (TSP) and a geothermal system (MGS) located in the volcanic arc.

The fore-arc resistivity structure exhibits typical resistivity values of more than 500 Ωm for the continental crust, where reduced values are associated with fault zones and young sediments.

A profile of long period MT stations will be conducted in the future to reach greater depths and therefore investigate the relation between the conductive features identified in this study and the deeper subducting slab and mantle structure. This profile will be extended into Argentina to better constrain the conductive anomalies located at Laguna del Maule.

## Additional files

**Additional file 1.** Apparent resistivity and phase curves.

**Additional file 2.** Dimensionality analysis.

**Additional file 3.** Sensitivity tests.

## Abbreviations

CC: Coastal Cordillera
CV: Central Valley
LdM: Laguna del Maule
MGS: Mariposa Geothermal System
MT: Magnetotellurics
PC: Principal Cordillera
SVZ: Southern Volcanic Zone
TE: Transverse electric
TM: Transverse magnetic
TSP: Tatara-San Pedro
