# Supplementary material for: Regional electrical structure of the Andean subduction zone in central Chile (35°–36°S) using magnetotellurics
Source: Earth Planets Space. 2017 Oct 12;69(1):142. doi: 10.1186/s40623-017-0726-z (PMC6961476; doi:10.1186/s40623-017-0726-z)
Supplement: Supplementary file 1 — Additional file 1. Apparent resistivity and phase curves. [file 40623_2017_726_MOESM1_ESM.pdf]

Additional File 1: Apparent Resistivity and Phase Curves

The apparent resistivity and phase curves of all sites used in the inversion are shown below. Observed data is shown in red triangles for TE mode and blue circles for TM mode. Preferred model response is shown in red and blue lines for TE and TM modes, respectively.

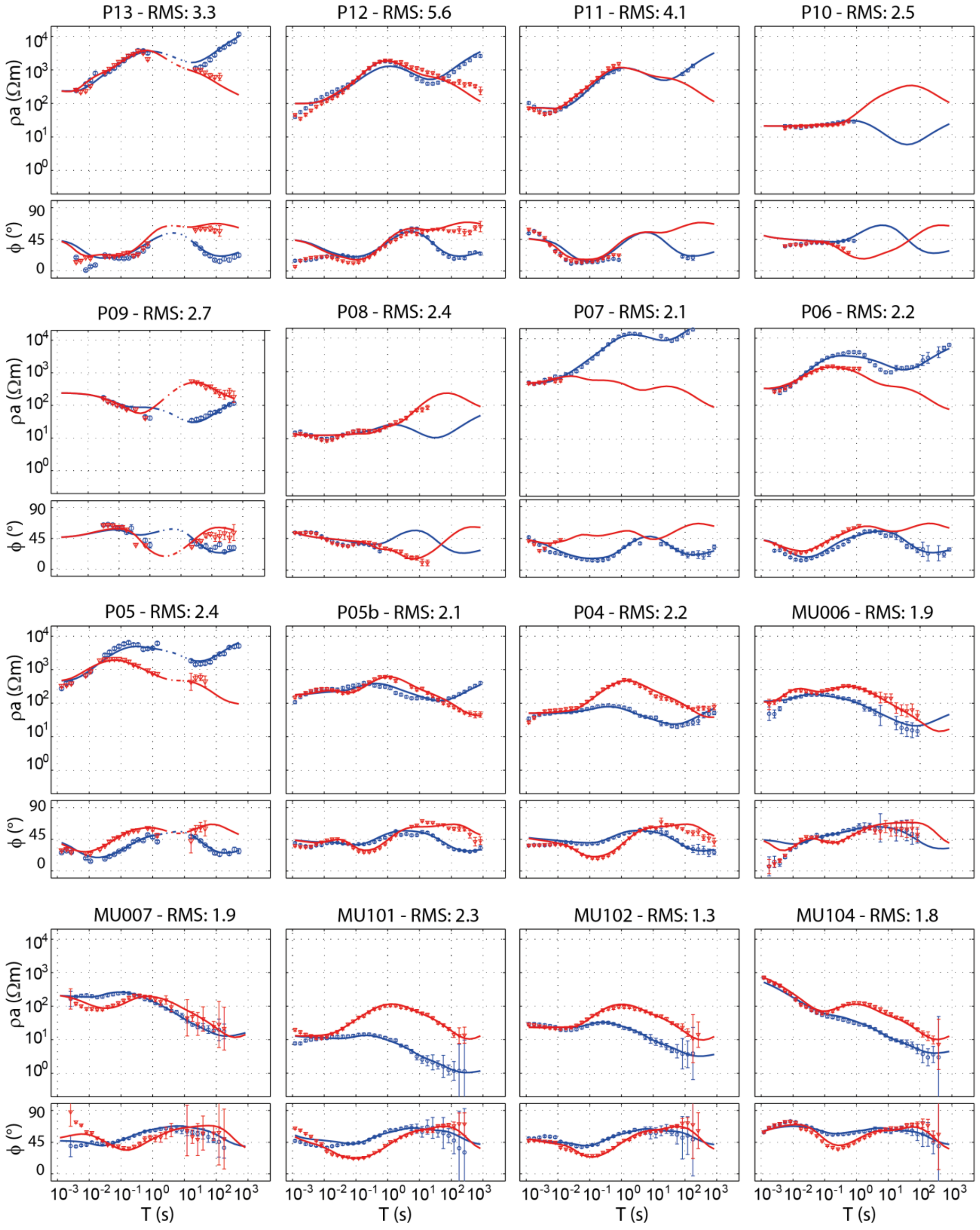

Figure 1.

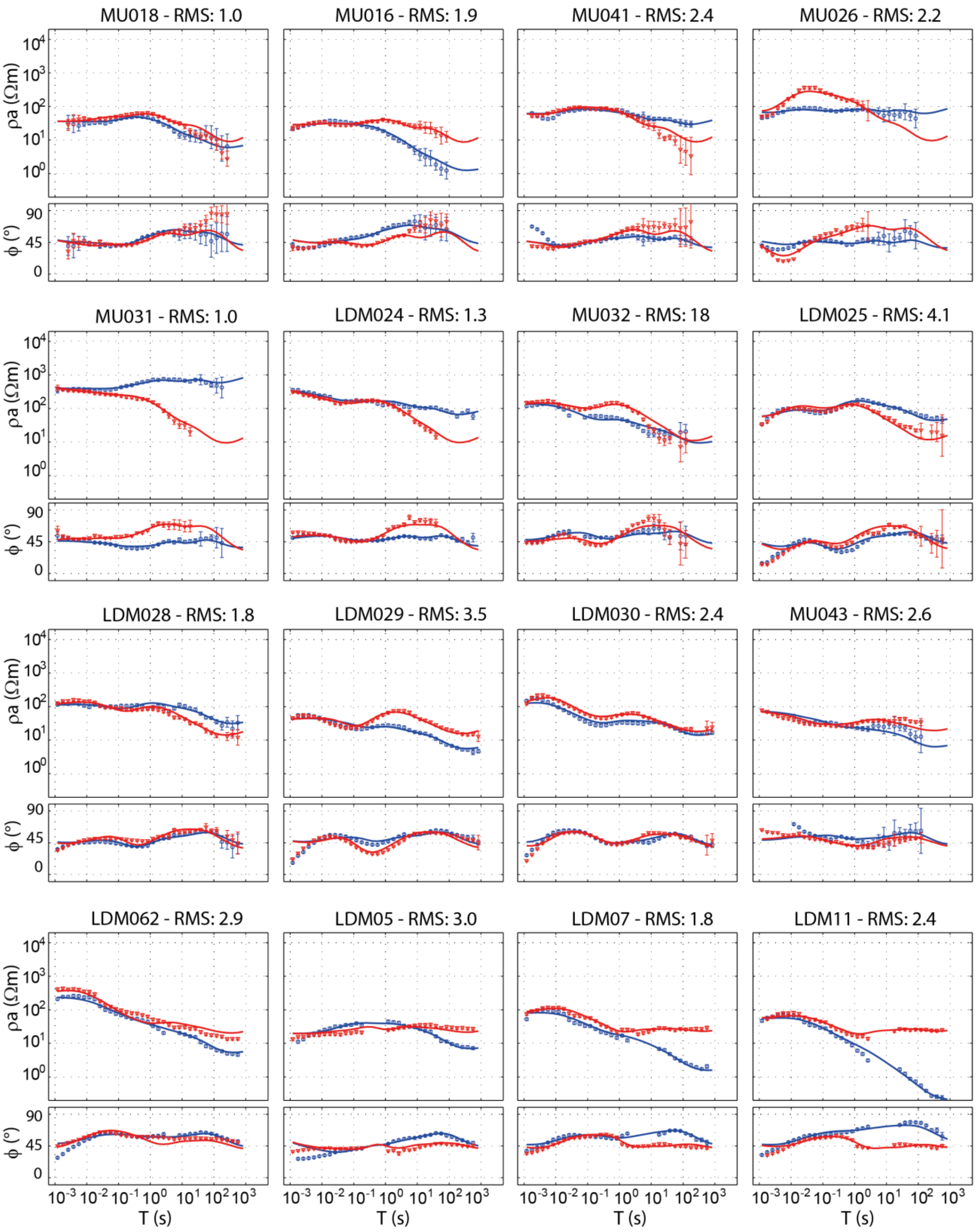
MU018 - RMS: 1.0
MU016 - RMS: 1.9
MU041 - RMS: 2.4
MU026 - RMS: 2.2
MU031 - RMS: 1.0
LDM024 - RMS: 1.3
MU032 - RMS: 18
LDM025 - RMS: 4.1
LDM028 - RMS: 1.8
LDM029 - RMS: 3.5
LDM030 - RMS: 2.4
MU043 - RMS: 2.6
LDM062 - RMS: 2.9
LDM05 - RMS: 3.0
LDM07 - RMS: 1.8
LDM11 - RMS: 2.4
ρa (Ωm)
φ (°)
T (s)

Figure 2.
