# Supplementary material for: Regional electrical structure of the Andean subduction zone in central Chile (35°–36°S) using magnetotellurics
Source: Earth Planets Space. 2017 Oct 12;69(1):142. doi: 10.1186/s40623-017-0726-z (PMC6961476; doi:10.1186/s40623-017-0726-z)
Supplement: Supplementary file 2 — Additional file 2. Dimensionality analysis. [file 40623_2017_726_MOESM2_ESM.pdf]

Additional File 2: Dimensionality Analysis

Induction Arrows

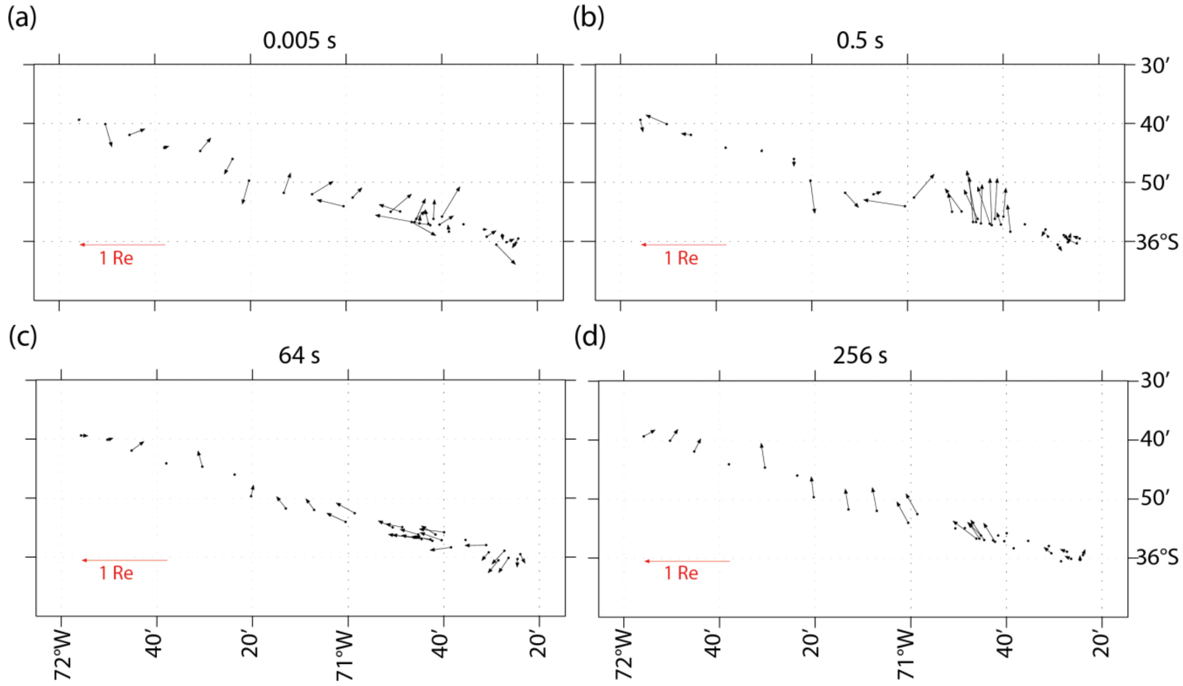

Figure 1.

The induction arrows were plotted according to Wiese (1962) convention for a few characteristic periods.

(a) For short periods, the arrows do not show any consistent pattern, probably due to local, shallow 3D features.

(b) The stations located at the volcanic front present arrows that point north, indicating a shallow conductor south of the profile. This is consistent with the low resistivity anomaly found by Hickson et al. (2011) interpreted as the alteration clay cap of the MGS. It is also consistent with a slightly higher skew value.

(c) Arrows located at the volcanic front and west point to the west, indicating conductors to the east along profile, while sites located north of the LdM present arrows pointing slightly to the south, indicating deep conductor anomaly at north.

(d) For longer periods the induction vector data is not consistent with the geoelectric strike as they have a large N-S component.

The results of the induction arrows are consistent with a volcanic arc with a more 3D structure than the rest of the profile.

Phase Tensor Ellipses

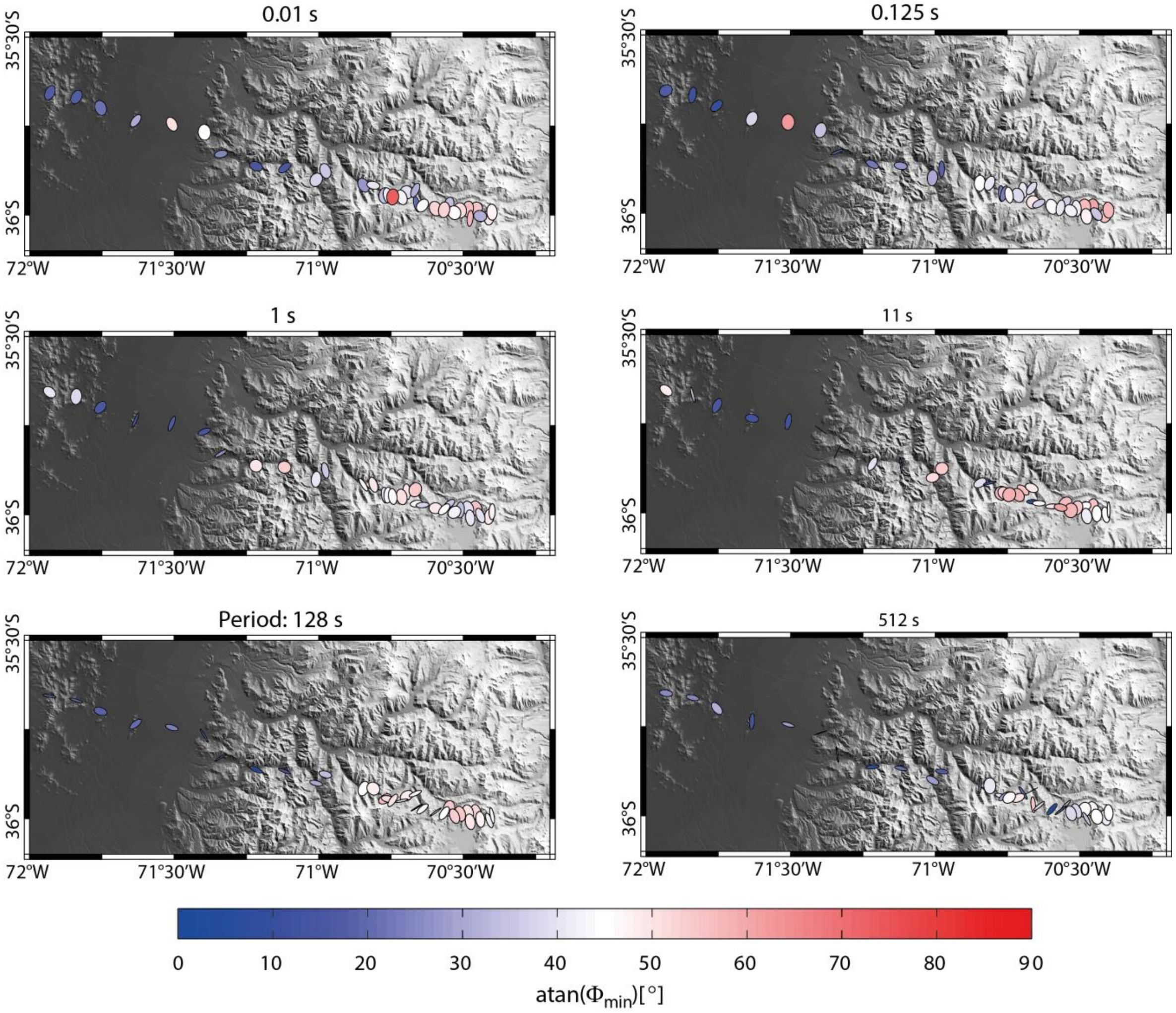

Figure 2.

Phase tensor ellipses according to Caldwell et al. (2004) were calculated as dimensionality indicators. For short periods, shape and direction of ellipses change clearly from site to site, presenting more flattening at the western side as period grows. For periods shorter than 100 s the stations to the west of the active volcanic arc present more flattening, but only for periods larger than 100 s ellipses tend to be elongated parallel to the profile direction.

From 1 second, one can observe differences in the behavior of the ellipses at the eastern side of the profile, with a minimum of the Phase Tensor larger than 45 degrees, while at the western border, these values tend to be less than 45 degrees. This behavior is in agreement with the presence of conductive features below the eastern part of the profile, as observed in the 2-D inversion results presented.
