# Supplementary material for: Regional electrical structure of the Andean subduction zone in central Chile (35°–36°S) using magnetotellurics
Source: Earth Planets Space. 2017 Oct 12;69(1):142. doi: 10.1186/s40623-017-0726-z (PMC6961476; doi:10.1186/s40623-017-0726-z)
Supplement: Supplementary file 3 — Additional file 3. Sensitivity tests. [file 40623_2017_726_MOESM3_ESM.pdf]

Additional File 3: Sensitivity Test

C1 Anomaly

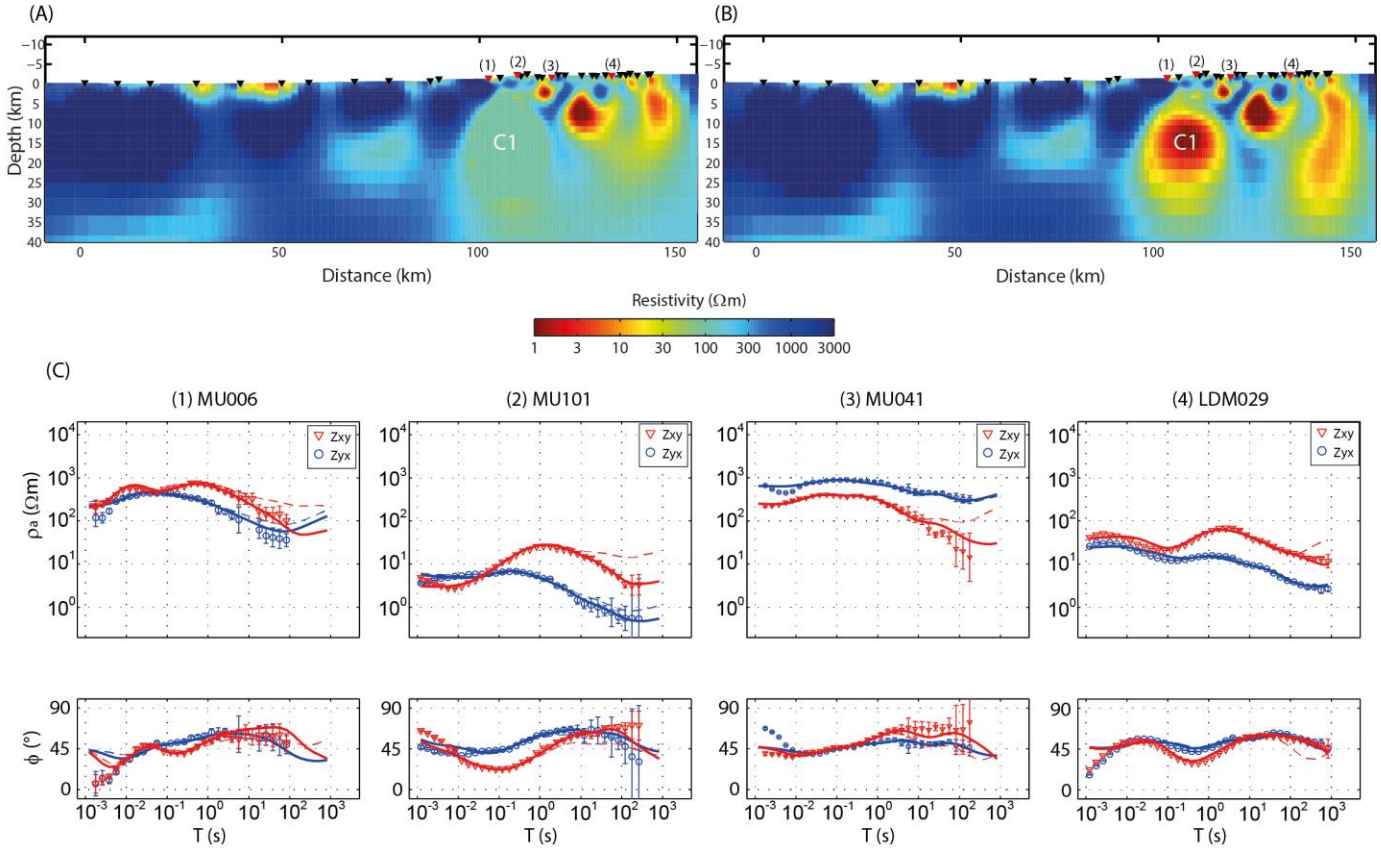

Figure 1

(A) C1 replaced by a 100 Ωm body.

(B) Inversion of the profile starting with model (A), conductive body C1 is recovered by the inversion after removing it.

(C) Apparent resistivity, phase and model response for several stations. The dotted line shows response from model (A), while the solid line shows the response from model (B).

The fitting decreases for both curves, apparent resistivity and phases, at mid-range to long periods for the sites near the anomaly. TE mode is more affected by TM mode, this is more visible for the phases.

C2 Anomaly

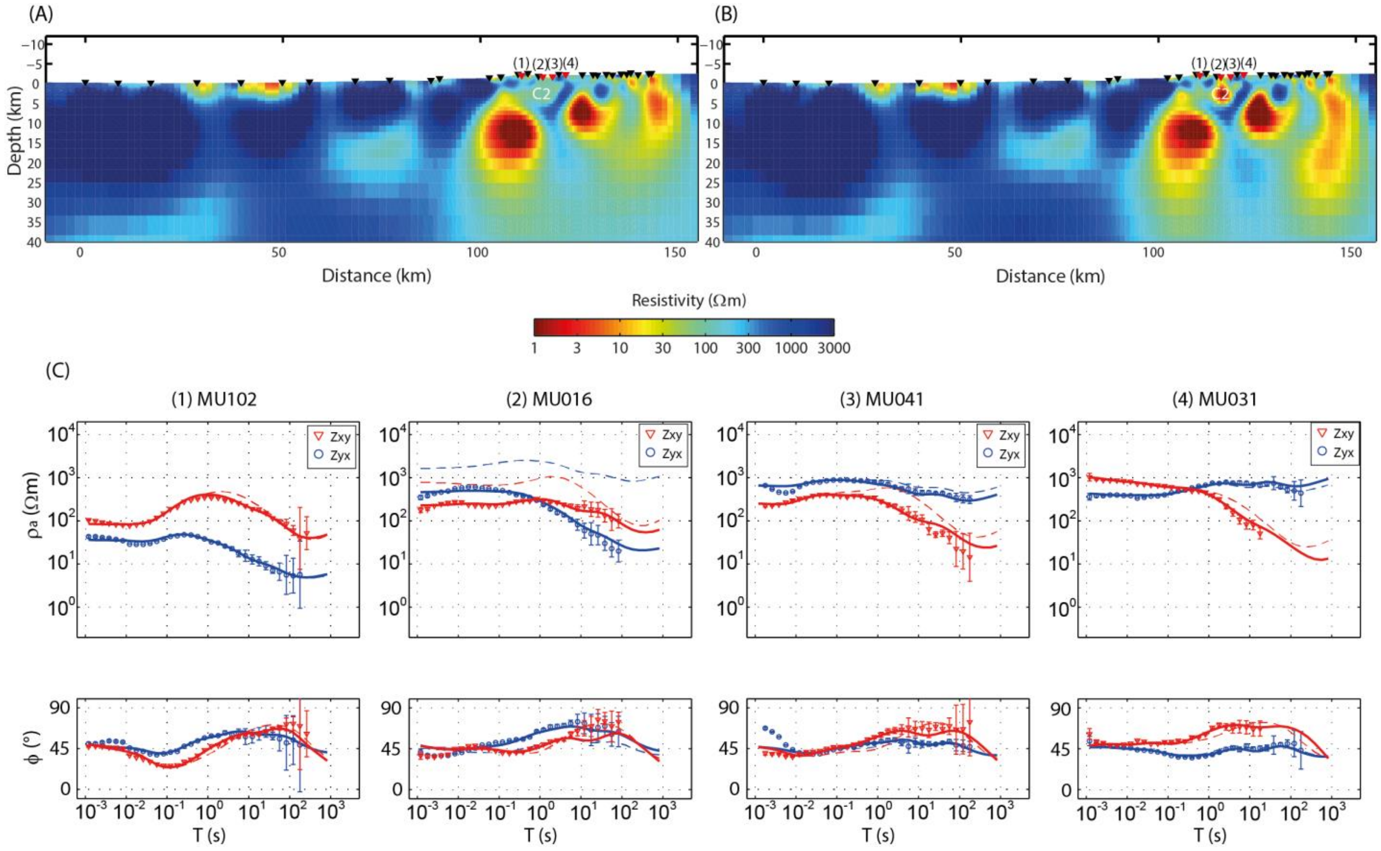

Figure 2

(A) C2 replaced by a 100Ωm body.

(B) Inversion of the profile starting with model (A), conductive body C2 is recovered by the inversion after removing it.

(C) Apparent resistivity, phase and model response for several stations. The dotted line shows response from model (A), while the solid line shows the response from model (B).

The apparent resistivity show great variations when C2 is removes, however this is not so clear for the phases. Nonetheless phases for sites close to C2 are also affected by its removal, for example the TM mode of site MU016.

C3 Anomaly

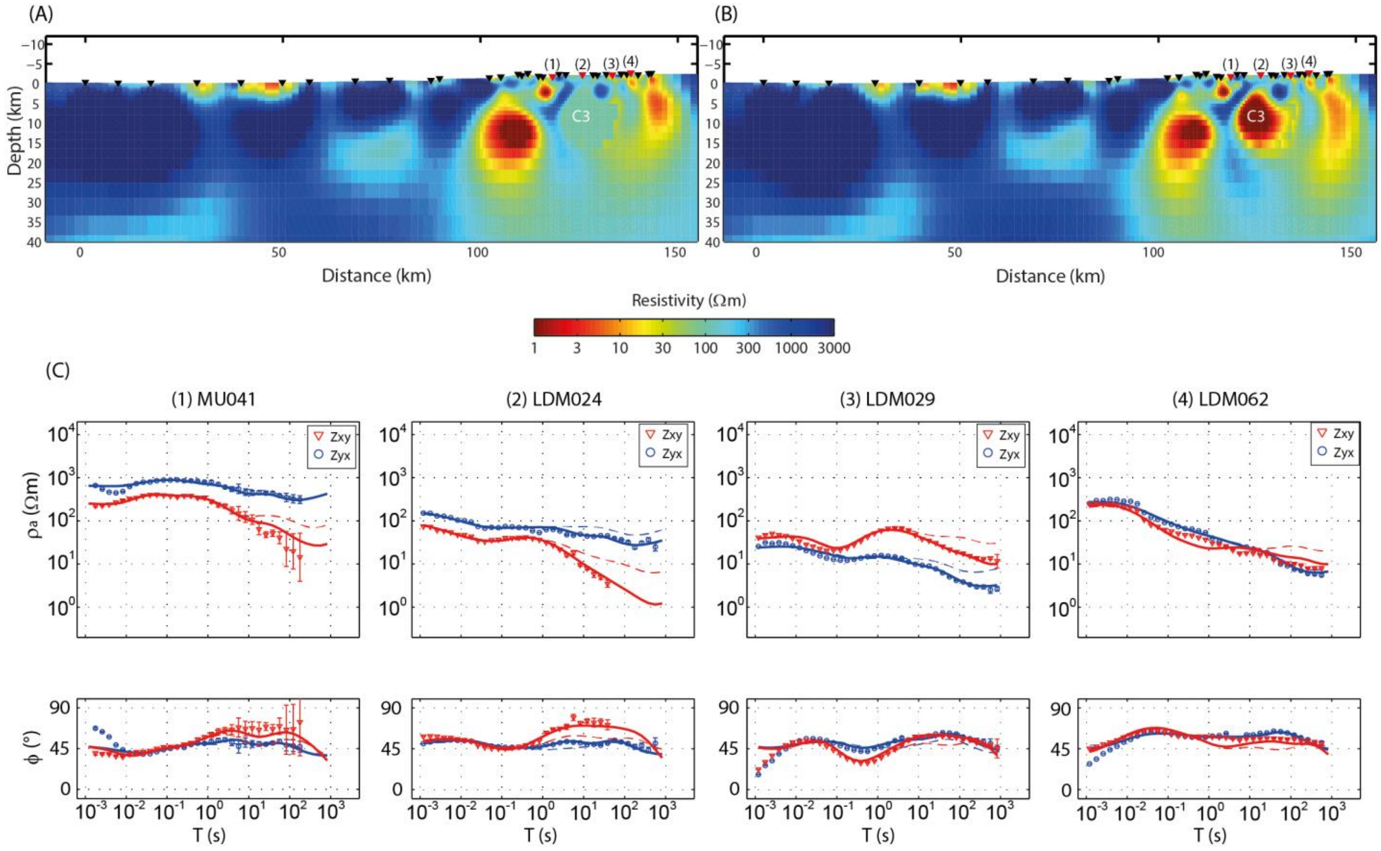

Figure 3

(A) C3 replaced by a 100 Ωm body.

(B) Inversion of the profile starting with model (A), conductive body C3 is recovered by the inversion after removing it.

(C) Apparent resistivity, phase and model response for several stations. The dotted line shows response from model (A), while the solid line shows the response from model (B).

The fitting decreases for both curves, apparent resistivity and phases, at mid-range to long periods for the sites near the anomaly. TE mode is more affected than TM mode, this is more visible for the phases.

C1, C2 and C3 as one conductive body

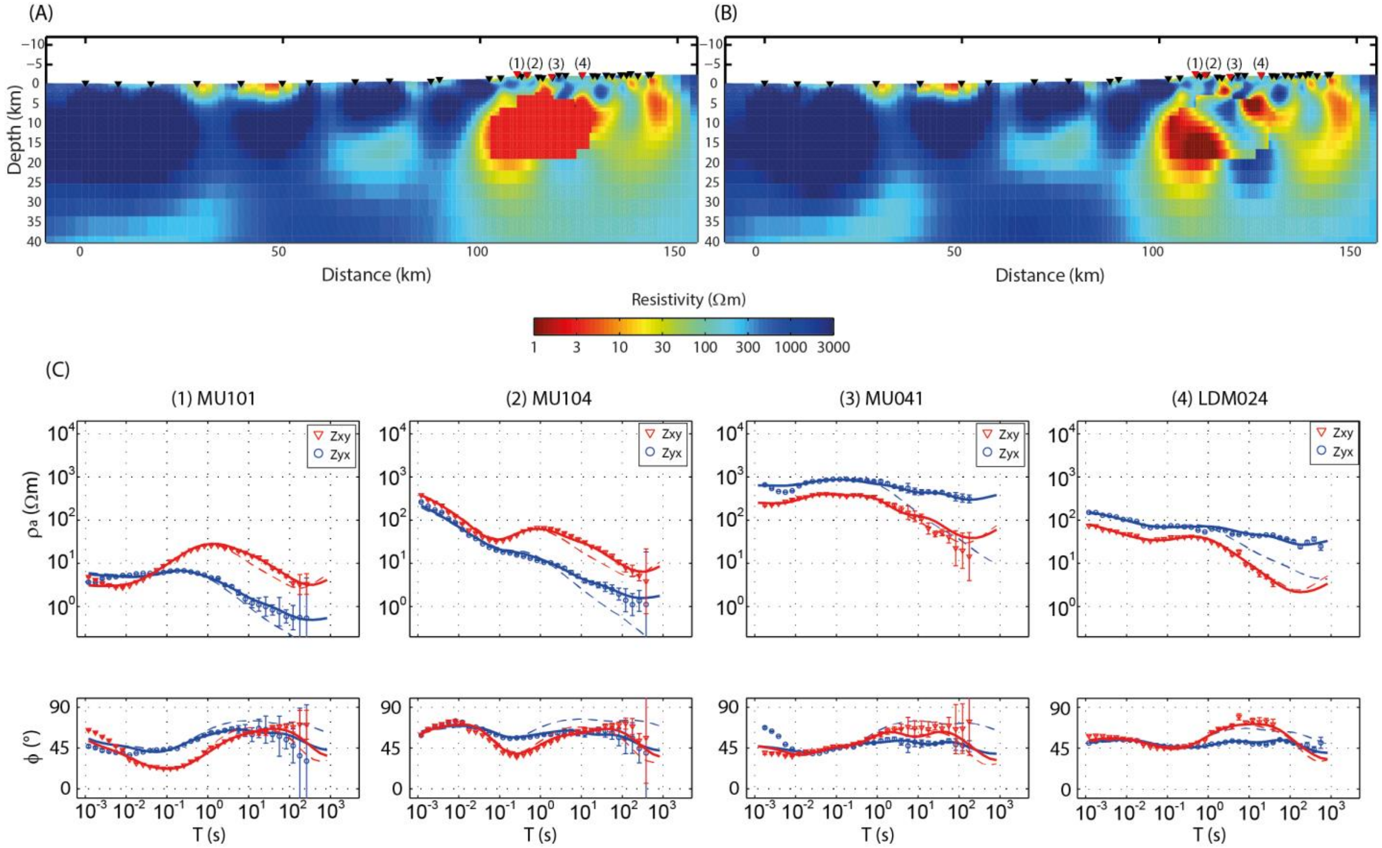

Figure 4

(A) C1, C2 and C3 where replaced by a single conductive body of 3 Ωm.

(B) Inversion of the profile starting with model (A). Even though the conductive bodies don't recover their exact previous shape, it is clear that a more resistive area is needed between the three conductors, suggesting that a single conductive body at the volcanic arc is not the case.

(C) Apparent resistivity, phase and model response for several stations. The dotted line shows response from model (A), while the solid line shows the response from model (B).

It is clear that for both curves, apparent resistivity and phases, the fitting decreases when a single conductive body is considered. TM mode shows the greatest difference, probably due to the effect of charges building up at lateral boundaries.

R1 anomaly

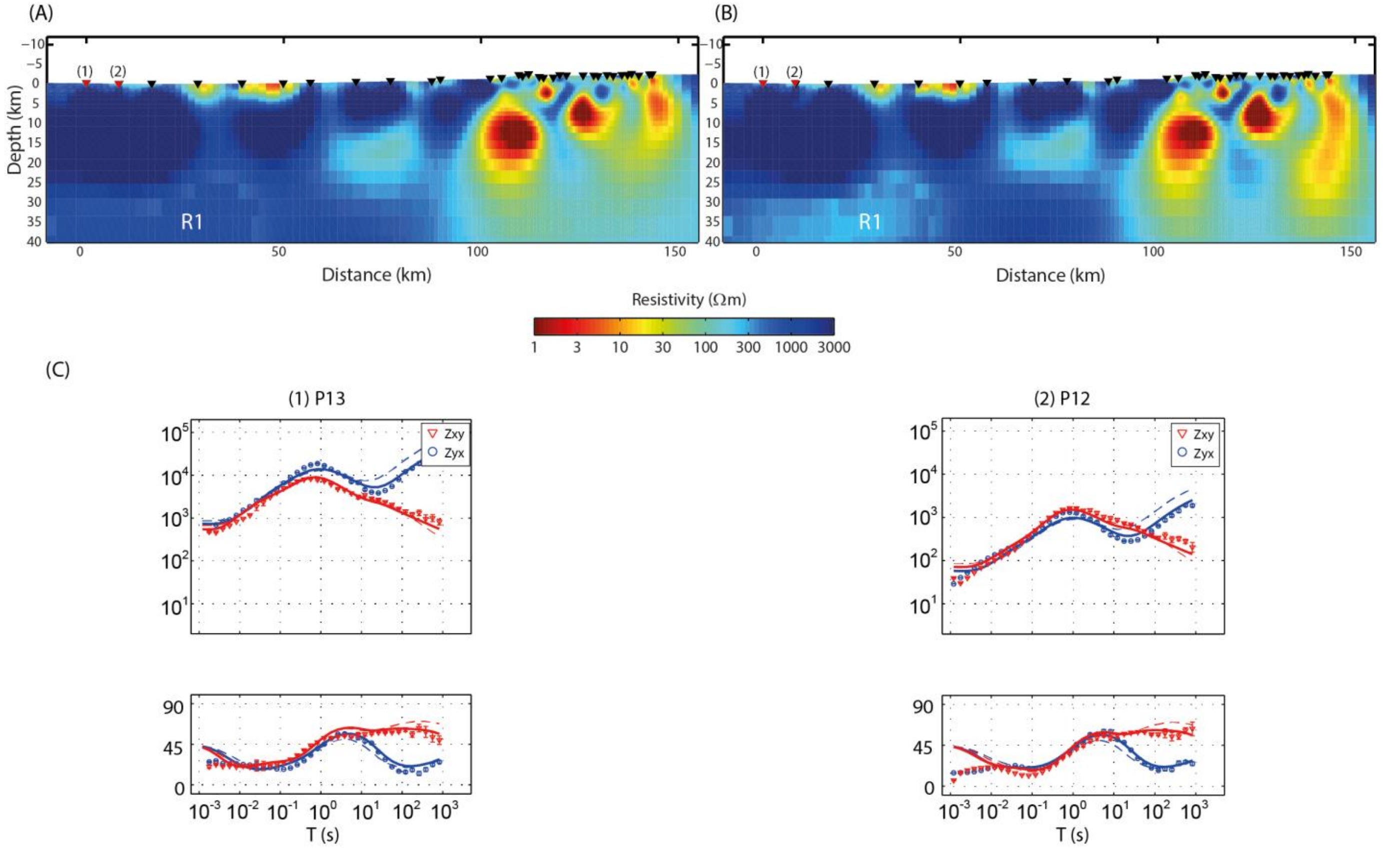

Figure 5

(A) R1 replaced by a 1000 Ωm body.

(B) Inversion of the profile starting with model (A), R1 is recovered by the inversion after removing it.

(C) Apparent resistivity, phase and model response for several stations. The dotted line shows response from model (A), while the solid line shows the response from model (B). The effect is only seen in two stations, because the ones located at the Central Valley don't have good data for longer periods, so it is no possible to compare both responses. Also the effect is not as important as when a conductive body is removed, because MT is more sensitive to more conductive anomalies.

The effect is most remarkable at longer periods of TM apparent resistivity, however it is also seen at mid-range frequencies of the TM phase and longer periods of TE phase.

R2 anomaly

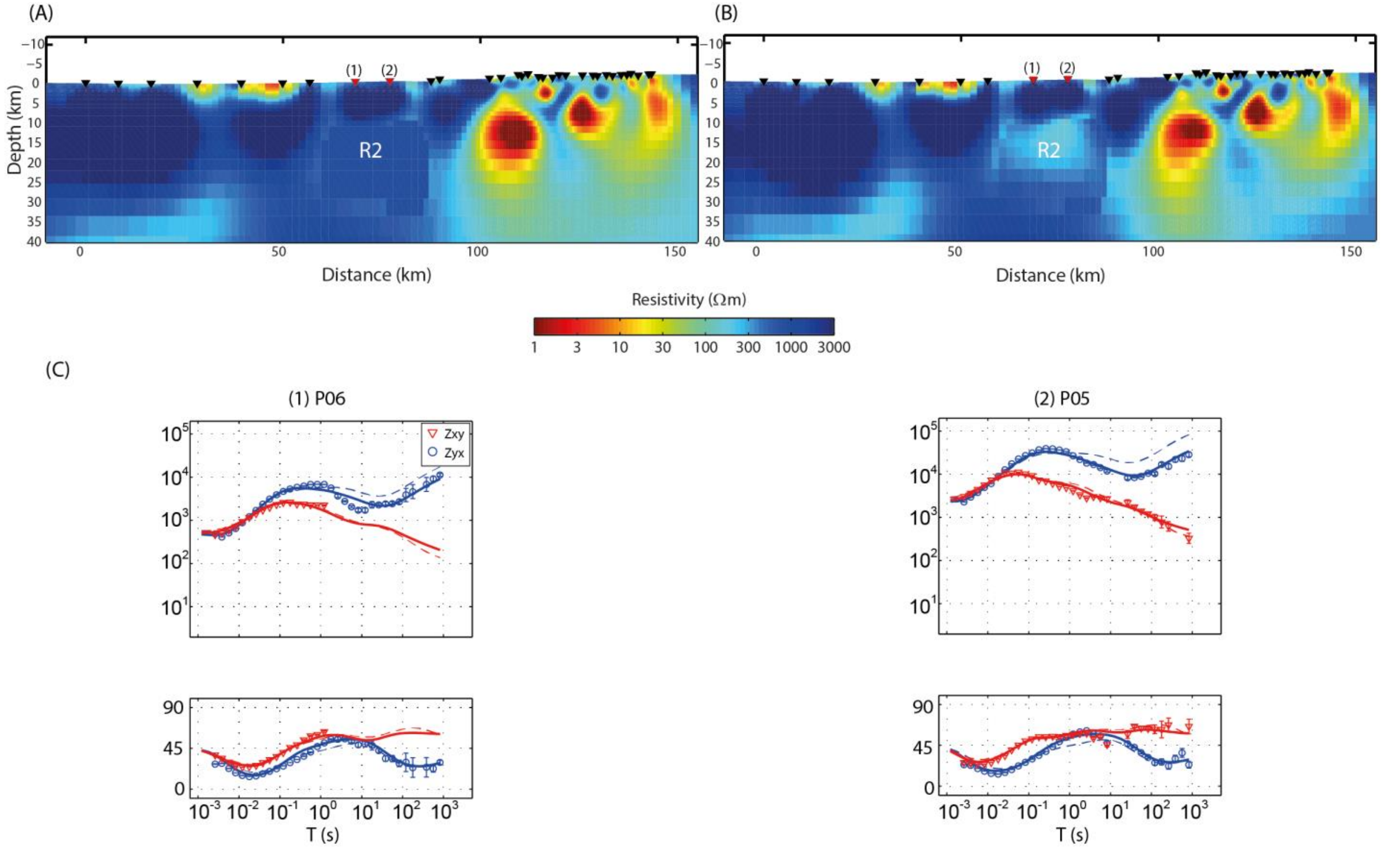

Figure 6

(A) R2 replaced by a 1000 Ωm body.

(B) Inversion of the profile starting with model (A), R2 is recovered by the inversion after removing it.

(C) Apparent resistivity, phase and model response for several stations. The dotted line shows response from model (A), while the solid line shows the response from model (B). The results are very similar to the one obtained after removing R1 in the way that the effect is not that visible as when a conductive body is removed.

The effect is most remarkable at longer periods of TM apparent resistivity, however it is also seen at mid-range frequencies of the TM phase.
